# Dentists’ perceptions on present and future dental practice during the COVID-19 pandemic: An embedded study

**DOI:** 10.12688/f1000research.109918.2

**Published:** 2022-09-20

**Authors:** Ramya Shenoy, Deeksha Das, Megha Mukherjee, Suprabha Baranya Shrikrishna, Ceena Denny, Violet D’Souza

**Affiliations:** 1 Department of Public Health Dentistry, Manipal College of Dental Sciences, Mangalore; Affiliated to Manipal Academy of Higher Education, Manipal, Karnataka, 575001, India; 2 Manipal College of Dental Sciences, Mangalore; Affiliated to Manipal Academy of Higher Education, Manipal, Karnataka, 575001, India; 3 Dept.Pedodontics and Preventive Dentistry, Manipal College of Dental Sciences, Mangalore; Affiliated to Manipal Academy of Higher Education, Manipal, Karnataka, 575001, India; 4 Department of Oral Medicine and Radiology,, Manipal College of Dental Sciences, Mangalore; Affiliated to Manipal Academy of Higher Education, Manipal, Karnataka, 575001, India; 5 Department of Dental Clinical Sciences, Faculty of Dentistry, Dalhousie University, Halifax, Nova Scotia, B3H 4R2, Canada

**Keywords:** COVID-19, Dentist, Dental Practice, Perceptions, Personal Protective Equipment

## Abstract

Background: The emergence of the COVID-19 pandemic has placed a significant burden on everyone. Although dental professionals are at an increased risk of COVID-19 infection, currently, very little is known about how oral health professionals and their professions could be affected by the pandemic. This study aims to investigate dentists' perceptions on present and future dental practice in light of the COVID-19 pandemic.

Methods: We conducted an embedded mixed-methods study at Manipal College of Dental Sciences, Mangalore, with Indian dentists registered with the Dental Council of India.

Results: Of the 976 participating dentists, 61% were females, 32% were 40 years of age or younger. Nearly half of the respondents (54%) acknowledged that the lockdown measures caused them a severe financial burden, and 56% were seriously concerned about being a source of infection to their family, friends, and community. Although 79% felt very comfortable or somewhat comfortable going back to work, they were all worried that Personal Protective Equipment (PPE) use would increase their financial burden and impact the number of patients seeking care. Even though a vast majority received the necessary information regarding returning to practice from their concerned dental regulatory bodies, some were unsure about the reuse of the PPEs because of the conflicting information they received.

Conclusion: The COVID-19 pandemic affected participants' professional lives negatively. Their major concerns were being a source of infection to their families and community. Providing information to dental professionals in a timely manner may prepare dentists to provide safe care to their patients while protecting themselves, their staff, and their families.

## Introduction

The emergence of the COVID-19 pandemic has placed a significant burden on everyone. In addition to the economic effects, healthcare systems and healthcare providers (HCP), whose work involves saving lives, faced an enormous burden. According to the World Health Organization (WHO), healthcare workers are people whose primary intent is to enhance health. They include doctors, nurses, paramedical staff, healthcare staff (administrative and support staff), and community workers. These are frontline workers who face a significant risk of COVID-19 infection and death.

With the rapid spread of the disease, many HCPs lost their lives while saving others. According to a systematic review, by May 2020, over 152,888 healthcare workers worldwide were infected, with 1,413 deaths reported. ^1^ The large majority of them (72%) were women, 51% were doctors, 39% were nurses, and 71% were men. In addition to the risk of COVID-19 infection, the pandemic also caused a significant physical and mental strain for healthcare workers. ^2^ Healthcare workers may continue to face the impact during and after the pandemic in the form of burnout, anxiety and depression, and post-traumatic stress disorders. ^3^ They may experience a loss of control, disruption of clinical practice routines, and fear of destabilizing health services. ^4^^–^^6^

Although most dentists are not considered crisis workers during a pandemic, many were required to provide emergency or urgent dental services. Many of these dental care providers may have faced challenges deciding what constitutes a true emergency. They were required to screen patients who required urgent dental intervention with questions based on epidemiological risk factors and COVID-19 symptoms. Overall, dental professionals are at a greater risk for cross-infection because of their proximity to the patient when providing care. Dental procedures, especially the aerosol-generating procedures performed on an infected person, produce debris that will eventually land on the dental personnel’s body, clothes, ^7^ and surfaces and the virus can remain viable for an extended period of time. ^8^ Also, exposure to the saliva of an infected person can result in the transmission of SARS-CoV-2 to the dental professional. ^9^ Lost working hours, the additional cost of personal protective equipment (PPE), and limiting aerosol-generating procedures have created financial pressure on dental practices. As a result, a large number of people working in dental clinics as dental assistants and dental technicians are equally affected due to the financial crisis. ^10^^–^^13^

Researchers in North America have observed a disagreement between their dental team members. ^14^ The disagreement was based on each of their staff members’ understanding and conception of the COVID-19 pandemic, its modes of transmission, and the risk of infection. As the dentists’ tasks increased, many of their team members experienced more significant work-related stress. Some worried about being an asymptomatic spreader of the virus to their family members, leading them to move away from their homes to protect their families. They also pointed out the possible economic recession affecting their job market, which can cause a financial burden for dental professionals. ^14^ A cross-sectional study that was conducted with 849 dentists in Italy reported that 80% of the participants were worried about the health of their families, 86% reported some income loss, and 94% feared a drop in the number of patients after the quarantine phase. ^12^

As the WHO declared the pandemic in March 2020, dental authorities in different countries developed guidelines to match their social, cultural, and environmental diversity. These recommendations demanded a massive change to traditional dental infection-control procedures. These modifications mandate using additional PPE, modifying the infrastructure of the workplaces, slowing the delivery of care, and limiting aerosol spread. These modifications can cause financial strain for dental care workers, including dental assistants, dental hygienists, and dental technicians. ^10^ Also, dentists may feel marginalized by the general public due to the nature of their profession. ^13^ Thus, the distress experienced by dentists during the COVID-19 pandemic is not limited to the risk of infection and getting the disease but is also modified by additional stressors such as social, cultural, and environmental factors.

Based on current estimations, the pandemic will prevail over months and will not disappear like earlier flu epidemics and is predicted to have periodic cluster outbreaks even after initial containment. ^14^ This increases the risk for dental health professionals carrying out dental treatment procedures as they cannot practice social distancing. There is also a risk of transmission through aerosol generation during treatment procedures. This could mean an additional burden to dentists in terms of PPE, disinfection, and measures to decrease the spread of aerosol ^15^ all of which will affect future dental practice. Currently, very little is known about how oral health professionals and their professions could be affected by the pandemic and there is very little guidance available to them at this time, thereby raising their anxiety and fear. Given that, we aimed to investigate the perception of dentists on present and future dental practice in view of the COVID-19 pandemic and to explore how prepared dental professionals are to provide dental care amid the COVID-19 pandemic.

## Methods

### Study design

We conducted an embedded mixed-methods study at Manipal College of Dental Sciences, Mangalore, with dentists registered with the Dental Council of India (DCI). The mixed-methods study included a cross-sectional study and a qualitative study using descriptive qualitative study methodology. The quantitative and qualitative studies were conducted concurrently between June 2020 and June 2021.

### Study population participants

*Our study participants were practising dentists and registered at the DCI.* The sample size was estimated for the cross-sectional study using the formula presented below with a 5% α and 5% margin of error.

$$n=\frac{{\left(\mathrm{Z\alpha}\right)}^{2}\mathrm{PQ}}{d^{2}}\;\;\;\;\;\;\;\;$$

where,

•
Zα = 1.96 (when α is 5% at 95% confidence limits)
• P = Assuming the proportion of the dentists’ concern for present and future dental practice (40%) ^12^
• Q =1-P (0.60)
• d = Acceptable margin of error (5%)

Based on this estimation, we were required to have 768 study participants for the cross-sectional study. We tried to reach the maximum number of dentists by posting the questionnaire link on WhatsApp and Facebook messenger. Participants were also encouraged to share the link with their networks. Therefore, we do not have data regarding how many people accessed the link to this study questionnaire.

### Ethical statement

Ethical approval was obtained from the Institutional Ethics Committee of Manipal College of Dental Sciences, Mangalore, Karnataka, India (Protocol No-20037). All participants were required to provide informed consent prior to participation in the study.

### Data collection

*Quantitative phase*

Data were collected using a newly developed study questionnaire that was developed by our research team of five public health dentists at Manipal College of Dental Sciences, Mangalore. A copy of the questionnaire can be found in the *Extended data.* ^24^ The questionnaire had 21 questions including the participants’ demographic details, area of specialty, their fear and anxieties towards COVID-19, financial burdens, the future of their practice, dental care delivery, and other updates concerning COVID-19. The content validity of the questionnaire was examined by the team members (RS and VD), and changes were made as required. The questionnaire was pre-tested with ten potential participants (dentists working in the dental colleges) who did not participate in the final study. Once pre-tested, the questionnaire development was finalized, and the questionnaire was created on Google Forms and pilot tested by the research team members.

Upon obtaining ethical approval, the survey link was sent to the potential participants through WhatsApp and Facebook messenger. The first author (RS) is a life member of the Indian Dental Association (IDA) and had access to the details of other IDA members. Other authors asked their networks to share the survey link across these platforms. The survey link also included study information (e.g., the purpose of the study, what is asked in the survey etc.) and an e-consent form that was required to be signed electronically. We also included a question to invite participants to the qualitative study. Those who were interested, were required to provide their contact details in the open text box provided in the survey.

*Qualitative phase*

The qualitative study included in-depth, semi-structured interviews with the participants who expressed interest in participating in this second phase of the study. Since this was a qualitative study, no sample size estimation was required, and thus, participants were recruited until we started receiving similar responses. Also, these participants were a nested sample of the quantitative study, who had provided electronic informed consent while participating in the survey, and thus did not need to provide additional consent for the in-depth interviews. All those who participated in the in-depth interviews were assigned a unique identification code. A total of eight participants were included in the qualitative part of the study. The in-depth semi-structured interviews were conducted using an interview guide prepared for the study. ^24^ Also, participants’ necessary sociodemographic information was collected to describe the general characteristics of the sample. The duration of the interviews was between 30 – 45 minutes. All interviews were conducted on the phone, and all interviews were audiotaped using a digital voice recorder (ICD-UX560F/B Digital Voice recorder, Sony India Pvt. Ltd) by the first author, who is a Public Health dentist with expertise in both quantitative and qualitative research. After each interview, the moderator summarized the content of the interviews to the participants and confirmed our understanding of the content. The participants were not contacted again once the interviews were completed. Since the data storage crashed after the analyses, we are unable to provide the transcripts of this study.

*Quantitative data analysis*

The survey data were analyzed using SPSS (27.0) version (IBM SPSS ^®^ Statistics), using 95% confidence (two-tailed). Differences in demographic variables were calculated using t-test for continuous variables and Chi-square or Fisher’s Exact test for categorical variables, depending on the distribution of categories. Mean scores and standard deviations for the outcomes were calculated.

*Qualitative data analysis*

The audiotapes of the in-depth interviews were transcribed verbatim. The transcribed data were read and reread by two investigators to gain a complete understanding of the content, highlighting the keywords. Data were labelled and color coded in Excel 2016, and the quotes were merged with the categories to describe the quantitative findings. The categories derived from the quantitative part of the study were the impact of the pandemic on the career, risk of infection, returning to work, practice environment and COVID-19 related information resources.

## Results

A total of 976 dentists responded to our survey invitation. Of them, 61.27%(n=598) were females and 68.44% (n=668) 40 years of age or younger, about 38.22% (n=373) were private practitioners, and about 56.76 % (n=554) were working at a dental college or hospital. Among those who had a spouse, 28.68% (n=280) had a spouse who was also a dental professional. A large majority of the participants 72.34% (n=272) did not have a study or practice loan of any kind. The general characteristics of the sample are presented in Table 1. The full dataset can be found under *Underlying data.* ^24^

**Table 1.** The general characteristics of the sample (n=976).

	n (%)
**Age**	
≤30 years	376(38.52)
31-40 years	292(29.92)
41-50 years	243(24.90)
51-60 years	61(6.25)
≥ 61 years	4(0.41)
**Sex**	
Male	378(38.73)
Female	598 (61.27)
**Has a dental professional spouse**	
Yes	280(28.68)
No	471(48.25)
Has no spouse	225(23.05)
**Number of years as a dentist**	
0-5 years	406(41.60)
6-10 years	186(19.06)
11 years of more	384(39.34)
**Current work status**	
Doing internship	49(5.02)
General dental practice	373(38.22)
Working in a hospital/Dental College	554(56.76)
**Type of dental specialty**	
Not a specialist (general dental practitioner)	569(58.30)
Orthodontist	79(8.09)
Operative dentistry/Endodontist	87(8.91)
Prosthodontist	69(7.07)
Periodontist	57(5.84)
Oral surgeon	30(3.07)
Pedodontist	41(4.20)
Public health dentist	44(4.52)
**Has outstanding study/practice loan**	
Have loan	272(27.87)
Have no practice loan	706(72. 34)

### Impact of the pandemic on the career

Six of ten respondents (65.27%; n= 637) reported that the COVID-19 pandemic negatively affected their professional life as a dentist, and nearly half of the respondents (n=529) acknowledged that the lockdown measures caused them a severe financial burden, although it did not affect their survival. That said, for one in ten respondents, the financial burden was very severe, affecting survival ( Table 2). About 46% (n=449) were worried that they might experience immediate financial loss leading to a financial emergency, and 10.14 (n=99) % feared additional PPE-related financial burdens they may have to bear upon resuming work once the lockdown lifted. This was also evident in our qualitative investigation. Most said they would follow the guidelines strictly and comply with the infection control protocol to provide safe care to their patients. Some expressed concerns that the cost of the PPE, additional equipment, and required infrastructure changes would add to the overall cost of treatment and thus, fewer patients would seek dental care, further constraining the dentists’ income. One participant expressed:

**Table 2.** The perceptions of the participants concerning returning to work.

	n (%)
**Impact of COVID-19 pandemic on the financial situation**	
I experienced a severe burden affecting my survival	108(11.07)
I experienced a burden, but it did not affect my survival	529(54.20)
Did not experience any burden	339(34.73)
**Greatest fears**	
Getting infected by patients	327(33.50)
Infecting my patients as an asymptomatic carrier	105(10.76)
Being a source of infection to my family, friends, and community	544(55.74)
**Getting back to dental work (dental care delivery)**	
I am very comfortable	165(16.91)
Somewhat comfortable	601(61.58)
Not comfortable	116(11.88)
Not sure	94(9.63)
**Collecting patients' travel history**	
Very important	687(70.39)
Somewhat important	191(19.57)
Not important	85(8.71)
Do not know	13(1.33)
**Checking patients' body temperature (screening)**	
Very important	581(59.52)
Somewhat important	266(27.25)
Not important	115(11.78)
Not sure	16(1.63)
**Willingness to provide care to patients presenting with flu-like symptoms**	
Provide care for using precautions following guidelines	106(10.86)
Perform emergency procedures only using precautions and following guidelines)	350(35.86)
Would ask the patient to get tested (for COVID-19) first	520(53.28)
**Preparedness to provide care to an individual who had a COVID-19 infection (but cured)**	
Very comfortable	171(17.52)
Somewhat comfortable	562(57.58)
Not comfortable	119(12.19)
Not sure	124(12.70)
**Perceptions about aerosol-generating procedures**	
Very dangerous	409(41.91)
Somewhat dangerous	460(47.13)
Very dangerous	89(9.12)
Not sure	18(1.84)
**Knowledge about emergency procedures**	
Not sure what dental emergencies are	174(17.83)
Explaining and convincing the patient that does not require immediate care	546(55.94)
PPE scarcity	99(10.14)
The physical structure of the practice	157(16.09)
**Disinfecting the operatory according to the guidelines**	
Well equipped	331(33.91)
Somewhat equipped (but need more information and resources)	547(56.05)
Not equipped	41(4.20)
Need more resources	35(3.59)
Not answered	22(2.25)
**Information needs**	
I received enough information	582(59.63)
I did not receive enough information	306(31.35
I received too much information	88(9.11)
**Satisfaction with the information and recommendations**	
Very satisfied	36(3.69)
Satisfied	282(28.89)
Somewhat satisfied	402(41.19)
Dissatisfied	155(15.88)
I do not know what to expect	101(10.35)

*Participant 00801:“Treatment will be expensive, more workforce, and we need to charge the patients. Patients may not come. Income will be less.”*

### Risk of infection

Given the nature of the profession, 55.74% (n=544) were seriously concerned about being a source of infection to their family, friends, and community. Nearly one-third of the respondents 33.50% (n=327) were concerned about cross-contamination, and 10.76% (n=105) were fearful of community spread, or being a non-symptomatic carrier. Forty-six percent (n=449) felt that they would have to spend more time on infection control procedures upon returning to work, and 39% (n=380) anticipated that they would have fewer patients than before the pandemic, thus affecting their income. These findings were reflected in our qualitative analyses as well. We observed respondents expressing anxiety about providing care. They were worried that there could be a scarcity of PPE and that the PPE may not be sufficient to prevent spread, and thus the risk of COVID-19 risk would remain high. Some raised concerns about patients’ records that were not electronic. Two respondents said:

*Participant 00803: “Universal precautions evolved through ebola, but even after wearing PPE cohorts of diseases coming out with COVID.”*

*Participant 00804: “Fomites- Patient files may act as fomites, carrying files from one department to another may spread the virus. They harbour for 16-24 hours. Digital files should be used if a patient is infected. Imagine the doctor treated a COVID patient; what happens if the records of that patient circulated?”*

Also, some raised concerns about the stigma against COVID-19. As a result, people were less likely to get tested. Such situations are seriously concerning. One participant said:

*Participant 00808: “People can hide the history. They avoid getting tested, and therefore, we cannot rely on travel history or triage them. Society has stigmatized COVID-19.”*

### Returning to work

About 78.49% (766) of respondents were very comfortable or somewhat comfortable going back to work, 13.94 % (n=136) were unsure, while a very small percent 1.95 (n=19) did not want to go back to work. A small percent (6%) were seriously concerned about returning to work and were considering changing their career path. When asked what modifications they would make to their work habits to protect themselves and their significant others and to prevent the COVID-19 spread, many expressed concerns about the patients’ travel history. A great majority 89.96% (n=878) considered it important or somewhat important to ask their patients about their recent travel history before providing them with dental care as recommended by the governing bodies. Also, 59.52% (n=581) felt that checking body temperature would also be very important. 53.28% (n=520) of the dentists said that if a patient reports flu-like symptoms, they would ask that patient to get tested first if they need to receive dental care. About 75.10% (n=733) said they would be comfortable providing dental care to patients who tested positive for the COVID-19 but with precautions. When asked to rate their distress level for providing care in a dental office setting to a patient exposed to COVID-19 on a Likert scale (scores range 0-10), 12% (n=117) had a score of 10, and 23% (n=224) had a score of 8, with a mean score of 6.93±2.20. Two participants who participated in the in-depth interview said:

*Participant 00804: “The first step is triaging the patients and having a good infrastructure.”*

*Participant 00806: “Private practice will be changed. Before, when we worked, no N95 masks and PPE kits. Now, we need to take small breaks, cannot work for a long time. No AC (air conditioner) and work efficiency will be decreased. Cannot use all the chairs (operatories).”*

### Practice environment

About 16.09% (n=157) were concerned about the physical structure of the practices where they worked, and felt that their practices were not ready to perform aerosol-generating procedures to provide emergency services. In such situations, 55.94% (n=454) expressed that they would educate their patients about non-essential dental care and convince them to postpone their non-essential dental care amid the pandemic. Around 41.91% (n=409) of the respondents felt that using aerosol-generating devices such as airotors and ultrasonic scalers was very dangerous at the time of the survey. About 17.83% (n=174) were unsure about what emergency dentistry was, while 10.14% (n=99) were concerned that there wasn’t adequate PPE available for dentists to deliver care safely. One respondent who participated in the in-depth interview was concerned about the risk of infection despite using the suggested filters and other guidelines:

*Participant 00805 “Patients are not allowed to spit in the spittoon for preventing direct spillage. The external devices do not take care of aerosol. The UV filters and extraoral suctions can be tried at least to minimize the aerosol to a certain extent. These just reduce 50% of aerosol, is it ok?”*

### COVID-19-related information resources

When we asked respondents whether they received the necessary information concerning returning to practice from their concerning dental regulatory bodies, 68.74% (n=670) said they received enough information, recommendations, and updates from the dental governing bodies or employers. About 32.58% (n=318) felt well equipped with information and resources, and 41.19% (n=402) were somewhat satisfied with the recommendations they received from their employers and respective governing bodies. In our qualitative analyses, we observed that few participants said that they received sufficient information to go back to practice while the others expressed a lack of clarity. Although most of them felt that N95 could be reused, some expressed concerns about reusing it. A few participants expressed:

*Participant 00802: “Very difficult, WHO does not know??? What about us? Is DCI (Dental Council of India) doing a good job?”*

*Participant 00803: “How can we decide reusing N95 mask? No evidence on reuse and sterilization … Maybe, wear a disposable mask on N95 and then reuse.”*

*Participant 00804: “We have to assume all patients are COVID positive. I don’t want to keep it and reuse it.”*

## Discussion

This embedded study was conducted with Indian dentists to investigate their perceptions on the present and future of dental practice in view of the COVID-19 pandemic and to explore their preparedness in providing dental care during the pandemic. To our knowledge, this is the first study of its kind conducted using both quantitative and qualitative research methods. We observed that a vast majority of participants felt that the pandemic negatively affected their professional life as a dentist, were afraid of being a source of infection to their families and community, and that 73.56% (n=718) were somewhat satisfied with the information they received concerning the return to work. That said, the majority of participants 61.58% (n=601) felt comfortable returning to practice.

In a similar study conducted in Italy during the onset of the pandemic, the authors reported that 75% of the study population said that COVID-19 had an extremely negative impact on their practice. ^5^ The proportion of those who reported a negative impact in our study was higher, revealing an urgent need to look into the matter closely. Also, 55.74% of our study participants feared that they would be a source of infection to their family, friends, and community, and such observations have been reported in other studies as well. ^5^^,^^6^ While evaluating the psychological distress in dentists, we found that a higher psychological tension pertained to the fear of getting infected with COVID-19 from a patient. ^5^ Ahmed *et al.* (2020) investigated the fear of dentists in over 30 countries regarding the COVID-19 pandemic. ^9^ They reported that the reason for their increased fear of transmitting the infection to families and friends was because of the prolonged incubation time before the development of symptoms and the duration of the virus thriving on various surfaces. Another legitimate fear among dentists, according to this study, was the repercussion of getting themselves and their families quarantined.

It has been found that COVID-19 remains aerosolized for three hours after contamination, and it can stay on plastic surfaces and stainless steel for up to two days. ^8^ This makes dentistry a high-risk occupation as working with airotors and ultrasonic scalers is a daily treatment method for most dentists. ^7^ In our study, around 41.91% (n=409) of the participants felt that using aerosol-inducing devices such as airotors and ultrasonic scalers would be very dangerous amid this pandemic. When providing the emergency services, 16.09% (n=157) believed their main concern was that the physical structure of the practice would not be ready for adequate aerosol control. Before the AIDS pandemic, dentists were not known to wear gloves, eye protection, or face masks, which now is a strict protocol in a dental set-up. Pandemics before have been known to revolutionize medical facilities. ^16^ There is a dire need to come up with a solution that can contain the aerosol contamination in a clinical set-up. Commercial air exchange devices and purifiers are being experimented with for dental clinics. ^11^ The idea may seem drastic, but it might be possible to have it as an accepted standard in a few years if COVID-19 persists in the long run.

A large majority of participants expressed concerns about the negative financial impact on their professional life due to the nature of their profession. About 46% felt that they would have to spend more time on infection control procedures to provide safe care to their patients and to protect themselves and their staff. About half of our participants (48%) reported that the lockdown did not affect their survival but caused a severe financial burden, as observed by Ahmed and associates. ^9^ A negative financial impact is expected as fewer people seek dental care during the pandemic due to lockdown measures, fear of getting infected, and their own financial strain. ^14^ Furthermore, 10.14% (n=99) feared that they would incur more expenditure for PPE kits. Along with shutting down the practice for several months and the cost of PPE kits and sterilizing their practices, the financial strain can lead to psychological deterioration, as was observed earlier. ^6^

WHO has therefore recommended various protocols on personal safety measures involving using an alcohol-based hand wash with increased frequency in a dental clinic along with using N-95 masks and PPE kits. ^8^ There are various other guidelines and protocols given by the Centers for Disease Control and Prevention (CDC) and WHO for dental practitioners and workers to help reduce the spread of COVID-19. ^17^^–^^21^ Despite these guidelines, our participants’ mean distress level was eight for risk of getting infected with COVID-19 from an infected patient in a dental practice set-up, which is alarming. Also, a significant number of the participants were not familiar with the guidelines. ^22^^,^^23^ Timely information resources provided to dental professionals could prove helpful. While such initiatives may exist, they have not yet reached practical momentum for Indian dentists. Hence it is important that the statutory bodies, such as the Dental Council of India (DCI), make it mandatory for dentists to attend training sessions regarding modifications in dental practice in the wake of COVID-19. Professional bodies like the Indian Dental Association (IDA) can be proactive in providing information regarding the guidelines.

The results can vary according to the general conditions of each country and how the country was affected, In India, one of guidelines given by DCI was to improve the ventilation of private dental clinics along with this maintaining the social distance were the biggest challenges Thus, the results of this should be interpreted cautiously and consider the following limitations. First of all, the study was conducted soon after the first wave of the COVID-19 pandemic and India was not significantly affected by the first wave compared to many developed countries. Although India suffers from a high population density and limited healthcare resources, people may have had a false sense of security believing that they have some sort of natural immunity, which protected the majority from getting infected during the first wave of COVID-19. This perception may have changed when India was severely affected in the second wave of COVID-19. Also, the study used a convenient sample of participants. Although we wanted to survey a larger number of dentists in India, we had a very low response rate. Furthermore, we did not use any validated scales, so the study results cannot be generalized to the dentist population of India. In the absence of valid scales, we utilized a mixed-methods research approach that helped us describe some issues related to dental practice in India that we may not have been able to describe otherwise. So present study has given a comprehensive picture on dentists’ perceptions on present and future dental practice during the COVID-19 pandemic.

## Conclusions

We observed that a vast majority of participants felt that the pandemic negatively affected their professional life as a dentist, that they were afraid of being a source of infection to their families and community, and that they were somewhat satisfied with the information they received concerning their return to work.

## Data availability

### Underlying data

Figshare: Dentists’ Perceptions on the present and future dental practice during the COVID-19 Pandemic. 10.6084/m9.figshare.19354835. ^24^

This project contains the following underlying data:

- covid-dentist 31-10.xls (description of data file)
- EXCEL-IMPORT-RS.csv (results of literature search)
  This project contains the following extended data: Questionnaire.docx (blank copy of the questionnaire and interview guide)

Data are available under the terms of the Creative Commons Zero “No rights reserved” data waiver (CC0 1.0 Public domain dedication).

The qualitative data related to this study is not available due to data storage crash. *This is documented in the status report of the research project and submitted to ethics committee*.

## Author contributions

All the authors did the search of the articles and review of the articles of the literature from various databases. RS and CD did the data collection. RS and VD developed and validated the questionnaire and analyzed the statistical data. VD prepared the tables. All authors contributed equally to the writing and editing of the manuscript. All authors read and approved the final manuscript.
