# Supplementary material for: Dentists’ perceptions on present and future dental practice during the COVID-19 pandemic: An embedded study
Source: F1000Res. 2022 Sep 20;11:453. Originally published 2022 Apr 22. [Version 2] doi: 10.12688/f1000research.109918.2 (PMC9623196; doi:10.12688/f1000research.109918.2)
Supplement: Peer review history

# Peer review: Reviewer response for version 2

**DOI:** 10.5256/f1000research.138709.r151619

**Published:** 2022-10-31

**Authors:** Santosh Kumari Agrawal

**Affiliations:** 1 Department of Public Health Dentistry, College of Dental Surgery, BP Koirala Institute of Health Sciences, Dharan, Nepal

- Abstract: Methods would be more elaborated
- Introduction: Please add a citation in the first paragraph (WHO)
- Main text method: how many dentists were registered at DCI and out of which how many had responded would give a clear idea about participants?
- Why Whatsapp and Facebook messenger apps were used instead of the registered email ids of dentists? 
- Is it the same questions used for both qualitative and quantitative methods? if yes, how can the same question assess the perception of the same person in terms of both qualitative and quantitative ways?
- Results: Please maintain uniformity while reporting n (00.00%). 
- t-test, Chi-square tests were used in this research, however, results regarding these tests have not been discussed.

Is the work clearly and accurately presented and does it cite the current literature?

Yes

If applicable, is the statistical analysis and its interpretation appropriate?

Partly

Are all the source data underlying the results available to ensure full reproducibility?

Yes

Is the study design appropriate and is the work technically sound?

Yes

Are the conclusions drawn adequately supported by the results?

Yes

Are sufficient details of methods and analysis provided to allow replication by others?

Partly

Reviewer Expertise:

Public health dentist

I confirm that I have read this submission and believe that I have an appropriate level of expertise to confirm that it is of an acceptable scientific standard.

# Peer review: Reviewer response for version 2

**DOI:** 10.5256/f1000research.138709.r150973

**Published:** 2022-10-04

**Authors:** Sumanth Kumbargere Nagraj

**Affiliations:** 1 Department of Oral Medicine and Oral Radiology, Melaka-Manipal Medical College, Melaka, Malaysia

Thank you for the clarifications. However, few of my concerns were not addressed.

1. Authors have used Google form link through social media. Here there are multiple concerns. Was this through a personal social media account or through the institutional social media account?
2. Google collects the data from any survey responses and thus cannot be termed as confidential. Here I am referring to the data access by Google which says that the data will be used by the company. Therefore, any survey data through Google doc cannot be called as 'confidential'. 
3. Drop out rate has to be calculated whether it is cross-sectional study (such as a survey here) or a clinical trial. 

It would be good to mention the details of Phenomenological analysis, such as type (transcendental, hermeneutic or existential; interpretive or descriptive) so that a reader can understand the influence of researcher on the data.

Is the work clearly and accurately presented and does it cite the current literature?

Yes

If applicable, is the statistical analysis and its interpretation appropriate?

Partly

Are all the source data underlying the results available to ensure full reproducibility?

Partly

Is the study design appropriate and is the work technically sound?

Partly

Are the conclusions drawn adequately supported by the results?

Partly

Are sufficient details of methods and analysis provided to allow replication by others?

Partly

Reviewer Expertise:

Systematic reviews, qualitative research, cross-cultural adaptation and clinical trials.

I confirm that I have read this submission and believe that I have an appropriate level of expertise to confirm that it is of an acceptable scientific standard.

# Peer review: Reviewer response for version 1

**DOI:** 10.5256/f1000research.121474.r135702

**Published:** 2022-05-09

**Authors:** Sumanth Kumbargere Nagraj

**Affiliations:** 1 Department of Oral Medicine and Oral Radiology, Melaka-Manipal Medical College, Melaka, Malaysia

I commend the author team to have conducted such a huge scale survey related to the COVID-19 pandemic.

 This study has a mixed-method design based on a newly developed questionnaire and a semi-structured interview guide. The questionnaire is developed by five public health dentists of the same institution and the authors have not reported results of validation. It is a good practice to share the results of all the validations conducted which thus makes the questionnaire development process transparent. 

 The first author had access to the details of other IDA members - Did the first author obtain the permission from the IDA to use the personal data of members? If so, the permission letter should be referenced. 

 Authors have used Google form link through social media. Here there are multiple concerns. Was this through a personal social media account or through the institutional social media account? Google collects the data from any survey responses and thus cannot be termed as confidential. 

 As the Google form link was shared through WhatsApp and FB messenger, it would be good to know how the authors controlled multiple replies by the same participant. Because of this problem, the authors could not get the data on total number of people who could access the link. 

 The authors have calculated a sample size of 768 based on 5% alpha value and 5% margin of error. However, it is good practice to consider 10 to 20% drop-out rate and include that in the sample size. 

 The qualitative data is collected from eight participants. Did the authors use the data saturation to stop the recruitment? It is not clear on the criteria for stopping the interviews with eight participants. 

 The qualitative data analysis described in the article has no mention about the method of analysis used. However, it appears that the authors have used 'thematic analysis' which needs to be mentioned. If yes, the authors who carried out the analysis should declare their onto-epistemological stand and how this influenced the analysis. 

 The authors mention that they were unable to provide the transcripts as the data storage crashed. It is not a standard practice to share the interview transcripts because of personal data protection laws across the globe. Therefore they need not be apologetic for not sharing the data. However, the data storage crash is an ethical problem which needs to be informed to their institutional ethics committee so that data storage issues are given importance in the future studies.

Is the work clearly and accurately presented and does it cite the current literature?

Yes

If applicable, is the statistical analysis and its interpretation appropriate?

Partly

Are all the source data underlying the results available to ensure full reproducibility?

Partly

Is the study design appropriate and is the work technically sound?

Partly

Are the conclusions drawn adequately supported by the results?

Partly

Are sufficient details of methods and analysis provided to allow replication by others?

Partly

Reviewer Expertise:

Systematic reviews, qualitative research, cross-cultural adaptation and clinical trials.

I confirm that I have read this submission and believe that I have an appropriate level of expertise to confirm that it is of an acceptable scientific standard, however I have significant reservations, as outlined above.

# Response

**Published:** 2022-09-14

**Authors:** CEENA DENNY

**Affiliations:** Manipal College of Dental Sciences,Mangalore,Manipal Academy of Higher Education,Manipal-575001, India

I commend the author team to have conducted such a huge scale survey related to the COVID-19 pandemic.

***Thank you sir for the suggestion. The authors appreciate your time taken to review this paper.***

 This study has a mixed-method design based on a newly developed questionnaire and a semi-structured interview guide. The questionnaire is developed by five public health dentists of the same institution and the authors have not reported results of validation. It is a good practice to share the results of all the validations conducted which thus makes the questionnaire development process transparent. 

***Ans-The questions asked were the general questions related to dental practice set up. So we have checked the content validity aspect of the validation. The detailed validation is the beyond the scope of this study.***

 The first author had access to the details of other IDA members - Did the first author obtain the permission from the IDA to use the personal data of members? If so, the permission letter should be referenced. 

***Ans-Being a life member of the Indian Dental Association , there is a separate Whatsapp group where members circulate the questionnaire pertaining to research and other important messages. This is agreeable by all the participants.***

 Authors have used Google form link through social media. Here there are multiple concerns. Was this through a personal social media account or through the institutional social media account? Google collects the data from any survey responses and thus cannot be termed as confidential. 

***Ans-The google form itself has consent form attached to it. Before going further, the participant can deny filling in at the first step itself.***

 As the Google form link was shared through WhatsApp and FB messenger, it would be good to know how the authors controlled multiple replies by the same participant. Because of this problem, the authors could not get the data on total number of people who could access the link. 

***Ans-As we have taken email id of the participants, there were no multiple entries.***

 The authors have calculated a sample size of 768 based on 5% alpha value and 5% margin of error. However, it is good practice to consider a 10 to 20% drop-out rate and include that in the sample size. 

***Ans-This is the cross sectional study, so we did not consider the drop out rate.***

 The qualitative data is collected from eight participants. Did the authors use the data saturation to stop the recruitment? It is not clear on the criteria for stopping the interviews with eight participants. 

***Ans-There is no sample size calculation for qualitative study. We used data saturation to stop recruitment.***

 The qualitative data analysis described in the article has no mention about the method of analysis used. However, it appears that the authors have used 'thematic analysis' which needs to be mentioned. If yes, the authors who carried out the analysis should declare their onto-epistemological stand and how this influenced the analysis. 

***Ans – We followed the phenomenological analysis. The significant statements were given codes and categories were derived and themes were formulated and phenomenon was explained.***

 The authors mention that they were unable to provide the transcripts as the data storage crashed. It is not a standard practice to share the interview transcripts because of personal data protection laws across the globe. Therefore they need not be apologetic for not sharing the data. However, the data storage crash is an ethical problem which needs to be informed to their institutional ethics committee so that data storage issues are given importance in the future studies.

***Ans-Thank you for the suggestion, About loss of data is documented in the status report of the research and submitted to Ethics Committee.***
